# Fusing concept to theory: identity fusion’s potential role in crime research

**DOI:** 10.1007/s11186-025-09637-z

**Published:** 2025-08-04

**Authors:** Martha Newson, Jack Cunliffe, Harvey Whitehouse

**Affiliations:** 1 Institute for Lifecourse Development, University of Greenwich, London, UK; 2 Centre for the Study of Social Cohesion, University of Oxford, 53- 55 Banbury Road, Oxford, OX2 6PE UK; 3 School of Social Policy, Sociology and Social Research, University of Kent, Canterbury, UK

**Keywords:** Desistance, Identity, Identity fusion, Turning points, Recidivism

## Abstract

Developing theories, evidence, and methods that could help to reduce crime is foundational to crime research. Here we present an interdisciplinary framework that can shed light on old theories and open up promising avenues for novel research into diverse criminogenic areas including violence, desistance, turning points, individual and family risk factors, and reintegration. This framework relates to ‘identity fusion’– a powerful form of group bonding whereby the individual’s personal and social selves become ‘fused’. We argue that the fusion mechanism is an underappreciated cause of– and simultaneously a potential solution to– many forms of criminal behaviour. Accordingly, we discuss applied opportunities to develop this approach from both theoretical and policy perspectives.

## Introduction

Identity fusion theory explains the most extreme of human behaviours that are perceived to protect or defend one’s group (Swann et al., 2009, 2012; Gómez et al., 2020). Described as a synergistic immersion of self in others, the fusion of identities evinces particularly powerful attitudes and behaviours that persist through life. Originating in social psychology (Gómez et al., 2011; Swann & Talaifar, 2018), many disciplines have helped to build understanding of this unique form of group cohesion, including developmental psychology (Reese & Whitehouse, 2021), anthropology (Sheikh et al., 2016; Buhrmester et al., 2022; Whitehouse & Lanman, 2014), evolutionary sciences (Whitehouse et al., 2017), and neuroscience (Bortolini et al., 2017; Apps et al., 2018). Fusion theory has generated a wealth of empirical studies establishing its association with extreme, self-sacrificial behaviours, including: engaging in extreme activism (Kunst et al., 2018), fighting with rivals (Newson et al., 2018), playing aggressively toward out-group members in video games (Vázquez et al., 2020), being willing to lay down one’s life in hypothetical scenarios (Swann et al., 2015; Bortolini et al., 2018; Newson et al., 2021), and even making the ultimate sacrifice and serving on the frontline in battle - be it warfare (Whitehouse et al., 2014) or gang-like violence (Newson et al., 2018; Chinchilla et al., 2022; Varmann et al., 2023).

As such, fused people should perhaps not be labelled as ‘criminal’, rather understanding *how* people become fused and *why* they behave in extremes could help inform best practice in designing interventions to decrease criminal behaviours. Despite studies related to criminal behaviour, surprisingly, the fields of forensic and criminal psychology are yet to embrace the identity fusion lens. We see potential for crime scientists to further develop identity fusion theory by applying it to real-world, high impact case studies. Our goal is not to critique existing theory in criminology but to integrate disparate areas of research that have hitherto been conducted in isolation. Bridging academic silos in this way can help with identifying blind spots, triangulating evidence relevant to solving common problems, and opening up novel research questions. This article adopts a two-pronged approach, addressing both the potential role of identity fusion in precipitating offending—thereby contributing to longstanding debates on the causes and onset of criminal behaviour—and the emerging potential of identity-based frameworks to inform contemporary discussions on desistance and pathways out of crime.

We propose that identity fusion theory is not only a helpful framework to understand the often self-destructive behaviours that crime science investigates, but also a way to explore how strong group ties could be harnessed for beneficial outcomes. The fusion framework may offer crime scientists opportunities to clarify or build on existing criminogenic knowledge, specific applications of which are discussed here. What we offer is not yet another individual risk factor to add to the plethora of trait variables most justice systems use; rather we present identity fusion as a more radical approach to understanding some key criminogenic behaviours and can thus be situated within the criminological landscape. Here we argue that to better understand certain aspects of the offending landscape, we need a lens that captures both individual-level differences and the group structures within which they are situated. We do not offer the psychology of identity fusion as a panacea for unresolved issues in crime science (or indeed propose that these can necessarily be resolved through interdisciplinary research). Instead, we see opportunities for researchers and practitioners working on crime to build on the fusion construct in relation to existing frameworks to better understand the extreme behaviours associated with group loyalty and the challenges of attempting to break such social ties. At the same time, we recognise the potential for interdisciplinary crime science to enhance fusion theory by drawing on criminological discussions around the subtleties of understanding extreme behaviours in a way that the theory has rarely previously received.

This article starts by outlining precisely what identity fusion is (and is not) and how the construct can be measured, before considering how it relates to established criminological work that could strengthen both research into identity fusion and the field of crime science.

## Defining identity fusion

### What is identity fusion?

Identity fusion describes the total overlap of personal and social identities; a visceral and irrevocable sense of ‘oneness’ or connection between an individual and a group (Swann et al., 2009, 2012; Whitehouse, 2018). In some cases, this extreme bonding may occur to another individual (Joo & Park, 2017; Walsh & Neff, 2018), a brand (Lin & Sung, 2014), a political ideology (Misch et al., 2018), and a value or moral belief system such as a religion (Fredman et al., 2017; Bortolini et al., 2018; Gómez et al., 2021). Fusion is theoretically distinct from group identification (Tajfel & Turner, 1979), a distinction that has been extensively empirically evidenced in the last decade (see Gómez et al., 2011; Bortolini et al., 2018; Gómez et al., 2019; and White et al., 2021), including in a meta study (Varmann et al., 2023). While the concepts of both identity fusion and group identification have their origins in Social Identity Theory (Tajfel & Turner, 1979), the idea that one’s sense of self emerges from group memberships, fusion was specifically developed to tackle enduring questions around extreme group behaviours, such as acts of terrorism (Swann et al., 2009), and has been extended to understand other group-based extreme behaviours including violent retaliation in ethnic or religious conflicts (Fredman et al., 2017; Ozkan et al., 2025), football violence (Newson et al., 2018, 2024a), or related to radicalized political ideologies (Ebner et al., 2024; Mason et al., 2025). Indeed, identity fusion is the strongest predictor of radicalisation among tens of different variables, many of which have been strongly supported in the past (Wolfowicz et al., 2021). We see a natural marriage between the rich theoretical developments and empirical methodologies offered by fusion theory’s explanation of extreme behaviours and criminology, the latter of which is in part devoted to understanding behavioural extremes.

Identity fusion is defined by four principles (Swann et al., 2012): the agentic-personal self; identity synergy; relational ties; and irrevocability, which are summarised in Table 1. In contrast to identification, where group identities alone lead highly identified individuals to enact progroup behaviours, highly fused people experience the simultaneous activation of both personal and group identities. Although fusion may wax and wane around a central point, research is yet to demonstrate how an individual might effectively de-fuse (Swann & Buhrmester, 2015).

**Table 1:** The principles of identity fusion, based on Swann et al. (2012), and their synergies with crime science

Principle	Explanation	Examples of potential synergies between identity fusion and crime science
Agentic-personal self	One’s personal identity is simultaneously activated when one’s group identity is activated.	Risk factors, particularly at the individual level such as how fusion might interact with impulsivity or empathy, and how these interact with other social controls.
Identity synergy	Both personal and group identities work together to motivate pro-group behaviours.	How group norms are internalised; how social learning theory can inform these processes; how the prosocial effects of fusion could be harnessed.
Relational ties	Other group members are also unique individuals.	Understanding why group identities persist after separation from the group; e.g., prison gangs.
Irrevocability	Fusion is stable; it is hard or impossible to de-fuse.	The process of desistance and the role that turning points or reintegrative initiatives can play.

Research on fusion and soccer fandom (or more specifically fan-related disorder) highlights a related, but distinct line of enquiry from the Elaborated Social Identity Model (ESIM) of crowd behaviour (Stott et al., 2008, 2020). The ESIM proposes that in anonymous group settings (e.g., mass sport events), depersonalisation increases the salience of social categories and group membership, and individuals are more likely to adopt behaviours in line with a salient group identity (Reicher, 2001).

The model has been extremely successful, even influencing policing strategies in Sweden (Stott et al., 2016, 2019). Applying this framework to soccer violence, research has shown how the perception of violence as a legitimate group behaviour among fan groups can change in response to police tactics, i.e., being treated as “hooligans” increases fans’ likelihood to adopt such a group identity and engage in group normative behaviours i.e., violence (see Stott et al., 2001; Stott et al., 2008; Stott & Pearson, 2007). This line of work has demonstrated considerable strength in explaining the interplay between group memberships and situational factors. Fusion theory complements the environmental, social and institutional contexts the ESIM covers, by explaining individual differences and why some individuals or subgroups are more challenging than others.

Identity fusion in its own right is neither good nor bad, right nor wrong. Instead, fusing to a group offers an opportunity for an individual to internalise the groups norms, attitudes, and a sense of collective strength. In this way, the fusion construct differs from traditional identity perspectives that present a more binary view of identity (‘good’ vs. ‘bad’ identities)– understanding that fusion to a group can be harnessed for group action that benefits the group (for better or worse). We pose that both/and thinking (Smith & Lewis, 2022) is the best way to regard fusion’s contribution to the literature; rather than identities being problematic or ameliorative, identities can be both.

As such, strong forms of pro-group commitment can find expression in both violent and peaceful courses of action. When ingroup loyalty promotes violence, it can do so in ways that are harmful not only to hated outgroups but to the enemy within. Moreover, the willingness of highly fused individuals to take one for the team means that they expect other members of the group to do that same. Although the harms sustained by ingroup members may seem inconsistent with strong forms of ingroup cohesion and outgroup hostility, both are logical outcomes of identity fusion. When personal and group identities become fused, outgroup threats are taken personally, motivating self-sacrificial behaviour to protect other members of the ingroup. This has frequently been shown using a measure of willingness to fight and die for the group which taps not only the individual’s readiness to kill members of the outgroup but also their preparedness to suffer harm for the group, even to make the ultimate sacrifice to advance its interests (Swann et al., 2012, 2014; Gòmez et al., 2020; Chinchilla et al., 2022; Varmann et al., 2023). The same willingness to risk life and limb for the group also explains the willingness of fused individuals to harm ingroup members who are perceived as a threat to the group or to accept their own willingness to endure harm out of group loyalty (Whitehouse, 2018).

### Measuring identity fusion

**Fig. 1:**
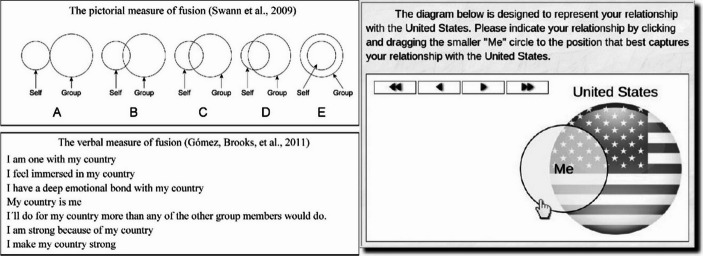
Three ways to measure identity fusion, adapted from Swann et al. (2012) and Jimenez et al. (2016)

Building on work from Social Identity Theory, measurements of identity fusion utilise pictorial, verbal, and digital tools. All three of the measures focus on total immersion of self in the group. The pictorial scale can be seen as a development of Aron et al. (1992)’s Inclusion of Self in Other scale, with an additional and final option for total inclusion in Other (or Group) (Swann et al., 2009 - see Fig. 1). This scale is ideal for use with language groups that have not yet had the verbal measure translated, with pre-literate or low-literacy groups, or in designs which test alignment to multiple groups to reduce the fatigue effects associated with longer scales. The pictorial measure was traditionally used as a binary variable (i.e., fused vs. not fused) (Swann et al., 2009; Whitehouse et al., 2014). Since the implementation of the verbal measure, it has become relatively common to treat fusion as a continuous variable or a gradient construct, if the data suggests that such use is appropriate. The verbal measure is a 7-item verbal scale, which captures more variation than its pictorial predecessor (Gómez et al., 2011- see Fig. 1). The verbal scale is more robust than the pictorial (Varmann et al., 2023) and the two correlate highly. The verbal scale has been translated into many other languages, including German, Spanish, Polish, Japanese, Indian, Norwegian, Portuguese, and Cantonese (Swann et al., 2014; Kunst et al., 2018; Bortolini et al., 2018). Finally, the DIFI or Digital Identity Fusion Index captures even more variation with two overlapping circles that participants can manipulate on devices including laptops, tablets, and phones (Jimenez et al., 2016). This makes the measure attractive to use in the field (provided there is Internet connection) or online studies, as it may be particularly engaging for participants.

### Pathways to fusion

Considerable research has been devoted to exploring the psychological pathways to fusion (Swann et al., 2012; Whitehouse & Lanman, 2014). Two pathways that have attracted particular attention are: (1) perceived shared biology, e.g., based on cues of phenotypic matching; and (2) the sharing of personally transformative experiences.

The shared biology pathway to fusion is exemplified by the so-called ‘brothers in arms’ mentality documented among frontline soldiers (Whitehouse et al., 2014) and may help to explain how people who are part of large, extended groups perceive their group ties in familial terms, from soccer fans (Newson et al., 2018) to American citizens (Buhrmester et al., 2015). Measures of perceived psychological kinship have been found to mediate the relationship between fusion and extreme self-sacrifice for both national and sporting identities (Buhrmester et al., 2015; Newson et al., 2021). Priming perceptions of shared biological traits has been found to increase psychological kinship, which in turn increases willingness to fight or die for one’s group (Swann et al., 2014). Finally, studies have shown that monozygotic twins tend to be more fused to one another than dizygotic twins (Vázquez et al., 2017a), and consequently, more willing to die for their twin (Tornero et al., 2017).

Second, the shared experience pathway to fusion has been investigated extensively in many groups, including war veterans, soccer fans, martial arts practitioners, family members, postpartum mothers, and members of fraternities (Whitehouse et al., 2017; Newson et al., 2016; Kavanagh et al., 2019; Tasuji et al., 2020). Shared experiences have been shown to produce an even stronger statistical effect on fusion than shared biology in a study of twins (Whitehouse et al., 2017). Taken together, this body of research suggests that intense experiences have the power to fuse individuals by creating an opportunity for experiences that are both personally transformative and group-defining to produce identity fusion. As dysphoric events stimulate more reflection than euphoric events, shared dysphoric experiences appear to be particularly prone to facilitate lasting identity fusion (Newson et al., 2016). This path to fusion has been proposed as offering a powerful framework to support social interventions to improve desistance from crime (Whitehouse & Fitzgerald, 2020), but to date research has not investigated in any detail how this might work in practice.

## Introducing identity fusion to crime science

Here we identify areas of crime science that identity fusion may help clarify or add weight to, posing questions that we believe could lead to fruitful future research and interdisciplinary collaborations, while trying to highlight some of the challenges and limitations of the approach. We consider these domains under two broad headings: (1) the origins of criminal behaviour; and (2) reintegration.

### Fusion and the origins of criminal behaviour

Identity fusion theory is of relevance to frameworks in criminology that help to explain the origins of criminal behaviour, such as differential association (Sutherland et al., 1992 [1947]) or its reimagining as social learning theory (Burgess & Akers, 1966), and social structure and social learning (Akers, 1998). Both frameworks explore how criminal behaviour is learned, explaining that the interaction between an actor and associates gives rise to knowledge, motivation and drives. These contribute towards ways of thinking and behaviours that are favourable or unfavourable to committing an offence. These definitions can in turn lead to action, which reinforces the learning, causing the process to loop with further reinforcement of behaviours as a result (Akers, 1998). Whether, and how, individuals fuse to criminal groups is clearly relevant to all these dimensions of social learning. Fusion is likely to impact not only the values and moral codes internalised by group members but the rate at which they are learned and the extent to which they are expressed in behaviour, building on existing research showing that fused individuals’ behavioural choices are influenced by the reasons for other group members’ behavioural choices (Paredes et al., 2018).

Classic labelling theory argues that deviance is not inherent in an act but by social reaction (Becker, 1963; Lemert, 1951). Once defined as an “offender,” an individual can fall into further (‘secondary’) deviance as the label becomes a master status that alters their self-perception, peer networks and their ability to negotiate their way through life (McAra & McVie, 2012). Identity fusion could clarify how this can accelerate. Being stigmatised by outsiders magnifies within-group solidarity: shared exclusion could be experienced as a personally transformative event that can enhance fusion (Newson et al., 2016; Whitehouse, 2018). The result is a reinforcing cycle where external condemnation amplifies fusion to a deviant group, which in turns leads to more deviant behaviour, or to a “deviant career”. Conversely, reintegrative approaches that allow the individual to accept the act without becoming (self) labelled as an offender and prevent stigmatisation (Braithwaite, 1989) should open space for fusion with conventional groups.

A related question is how identity fusion interacts with known risk factors for offending. At the within-individual level, known risk factors are reviewed extensively (e.g. Farrington, 2007; Farrington & Welsh, 2006; Jolliffe & Farrington, 2010) and include: a measure of intelligence; personality; agreeableness and/or conscientiousness; temperament, usually operationalised as irritability, low amenability and adaptability; empathy; impulsivity; self-control; and morality. Previous work has explored the interaction between morality and fusion, finding that individuals who reported fusion to their families or country were more likely to report that they would engage in violent behaviour to protect their group in the future, but only if they already had a moral belief in the justifiability of violence (Chinchilla et al.,^2022^). More generally, the effect of fusion on violent group behaviours is moderated by age and gender, such that younger men are particularly likely to engage in extreme pro-group behaviours that may include violence (e.g., gang rivalries) (Varmann et al., 2023); with the exception of being under conditions of group threat, when older males also become more violent, at least among British soccer fans (Newson et al., 2022).

There remain, however, multiple under-explored individual-level risk factors. Impulsivity, for instance, is a central determinant of anti-social behaviour and offending (Jolliffe & Farrington, 2006; White et al., 1994) and low cognitive empathy was found to be strongly related to offending in a 2004 meta-analysis (Jolliffe & Farrington, 2004), with the strongest relationships for violent offending and younger people. Tentative evidence suggests that identity fusion can lead to greater empathic concern, and that this in turn leads to more self-sacrifice (Landabur et al., 2022), while impulsivity can lead to more pro-group extreme sacrifice for romantic relationships in fused people (Joo & Park, 2017). Nonetheless, there is a pressing need for a greater understanding of how fusion interacts with these criminogenic variables. For instance, how might fusion restrain impulsivity for the sake of the group? Does a fused individual feel greater empathy for their own group and how might this affect criminogenic behaviours?

Closely related to the question of impulse regulation is the concept of ‘control.’ Control theory suggests that bonding with a law-abiding group could deter deviance (Piquero et al., 2010). Given that fusion is a particularly strong form of group bonding it could serve as an exceptionally strong control agent. Nonetheless, it is worth noting that some proponents of control theory, particularly as articulated by Hirschi (2002), reject the conceptualization of ‘social bonds’ as meaningful ties among those engaged in deviance. From this perspective, individuals involved in antisocial conduct are fundamentally incapable of sustaining genuine social attachments, consistent with the notion that there is ‘no honor among thieves’. This position stands in marked contrast to differential association theory (Sutherland et al., 1992 [1947]) and extensions within social learning theory (Akers, 2017), which posit that deviant behavior is often a product of group dynamics, wherein individuals acquire definitions favorable to law violation through sustained interaction with deviant peers. At the heart of this disagreement lies a sequencing question: does group membership precede and facilitate delinquent behavior, or do predisposed individuals selectively affiliate with delinquent groups? Fusion theory would suggest that groups themselves cultivate these behavioral outcomes (Swann et al., 2012; Whitehouse, 2018; Gómez et al., 2020), perhaps aligned with social learning theorists– while control theorists are likely to assert that those with low self-control gravitate toward similarly predisposed peers (Gottfredson & Hirschi, 1990).

Integrating insights from the control-learning debate may thus be crucial for clarifying the causal pathways linking fusion, group membership, and antisocial or prosocial action. For instance, previous research has shown that fused individuals can commit violent acts to protect their groups, perhaps indicating the potential for negative consequences associated with this extreme form of bond. But fusion may just as plausibly influence the expression of self-control in nonviolent ways. Tittle’s (1995) control balance theory posits that both control deficits and surpluses can lead to non-conforming behaviour. However, with fused individuals feeling immersed in their group, control balancing may take on a new dimension heavily influenced by the reciprocal nature between group and self for highly fused individuals, aligning with Paternoster’s (2017) call to treat offending as an intentional act shaped by meanings that actors actively construct rather than mere ‘happenings’: Do fused people feel they control their group, or the other way around?

In particular, General Strain Theory (GST) focuses on the motivation (typically anger and resentment) that causes action as a coping mechanism, but not whether that action is positive or negative– some may choose legitimate, productive responses whereas other may choose illegitimate, harmful ones (Agnew, 1992, 2006). Because fusion increases the attachment to group norms and frameworks, it can therefore condition how actions are undertaken. When a fused individual is embedded with a law-abiding group, strain-induced anger/resentment may be directed towards pro-social sacrifice such as volunteering or protesting to ‘right wrongs’, whereas fusion to illegitimately oriented groups might magnify the feeling into retaliatory or criminal action. In GST terms, fusion acts as a filter that directs the response to the strain, via emotion, towards either positive or negative behaviour. This leads to the testable hypothesis: that strain will predict criminal responses only when the person is fused to deviant groups that normalise criminal behaviour, and vice versa.

Just as important in understanding the origins of crime are higher-level risk factors (see Farrington and Welsh (2006) among others), including family criminality, large family size, child rearing methods, parental conflict and parental substance use. Swann et al. (2014, p. 912) found that most people are fused to their family but there remains a question as to how understandings of identity fusion could expand on the mechanisms through which familial risk factors influence offending behaviour. Importantly, what characteristics of fusion might lead to deleterious familial effects, and under what conditions? How do such effects compare to the consequences of an absence of family fusion, considering the overrepresentation of care-leavers in the criminal justice system? An understanding of these processes and how familial fusion, or a lack thereof (Whitehouse & Fitzgerald, 2020), can activate a willingness to act in societally harmful ways could thus illuminate core areas of crime research.

From a developmental perspective, fusion to close family is expected to start at around age one as part of healthy attachment and processing cues of facial similarities (Reese & Whitehouse, 2021). This occurs not only between child and parents, but also extended family, which serves to ‘phenotypically match’ individuals in one’s family. Nonetheless, the process of fusion to family is not inevitable, i.e., the child must be exposed to family members. As such, growing up without a family under the age of one, would be considered detrimental to family fusion. Without this early fusion to family, fusion to more extended targets, such as friends or even society as a whole, may become problematic as the individual will lack a secure base from which to develop other healthy group attachments (Klein & Bastian, 2022).

Relatedly, the fact that fused individuals may project familial ties onto extended groups (Swann et al., 2014), could help explain fusion within tight-knit criminal groups, such as mafia organisations or criminal gangs. Although research on fusion with criminal groups is limited, it is theoretically plausible that some individuals stay loyal to law-breaking groups because they are fused to them, despite the personal costs of prolonged imprisonment that may follow from those loyalties (Rosenfeld et al., 2003). Fusion may also help explain ongoing attachment to prison gangs once the individual has been released (Federal Bureau of Investigation, 2011; Pyrooz & Decker, 2019). Once individuals perceive other group members as comprising the same essence as themselves, whether through a process of sharing experiences or feelings of psychological kinship, it may be difficult to de-fuse and leave the group behind. Furthermore, despite an absence of law-abiding values, such groups may confer supportive and wellbeing effects, simply by virtue of feelings of belonging (Haslam et al., 2009; Tunçgenç et al., 2023).

There is some evidence for low levels of fusion to positive social groups and high levels of fusion to criminal groups among formerly incarcerated individuals in Australia (Whitehouse & Fitzgerald, 2020) and Jihadists serving prison sentences in Spain (Gómez et al., 2021); as well as links to reoffending behaviours for formerly incarcerated people fused to negative social groups in the UK and US (Newson et al., 2024b). Australian former-prisoners reported much lower levels of fusion to family than is typical of the general population in most countries (Swann et al., 2014; Varmann et al., 2023). This absence of fusion to family may indicate that interventions are required to help build family or family-like relationships, which are vital to in turn allow for fusion and loyalty to broader social categories such as one’s community or the state.

Low levels of fusion to family may indicate a lack of social buffers in times of stress, contributing to reduced wellbeing and impaired decision making (Tunçgenç et al., 2023). This is not to say that all people should be encouraged to fuse with their families since some families are likely to be harmful to one’s mental or physical wellbeing. Nevertheless, a lack of kin-like structures may limit development of socially encouraged behaviours such as loyalty, honesty, and co-operation. Having a secure base from which to form healthy attachments may help to cultivate self-sacrificial behaviours and good citizenship that contribute to a thriving and co-operative society. How can young people who grow up in care be best supported to develop family-like fusion to reduce the risks associated with this demographic group? In what ways could fusion to families who proliferate criminogenic values be harnessed to encourage positive behaviours by family members seeking to avoid or desist from crime?

Different forms of fusion will likely contribute to different kinds of offending. For instance, Chinchilla et al.’s (2022) work focused only on violent offending in cases where group coherence is threatened in the Spanish context, while Newson (2019) analysed the role that fusion can play in escalating the violence associated with soccer hooliganism in Brazil. Most of the fusion research cited here has focused on violent offending, or on acts of self sacrifice and other extreme behaviours. Pending enquiry, there is currently no identity fusion work that we know of that analyses offending behaviour other than violence, such as fraud, computer misuse, sexual offending or acquisitive crime, and it may be the case that fusion only interacts with violent offending, as has been seen and noted above.

It is, however, possible that there are forms of fusion that could lead to escalation in non-violent forms of offending, or indeed that fusion to the right targets could effectively act as a brake on these behaviours. Particular examples are the role that fusion might play in forming online communities, such as the much discussed ‘incel’ groups (Tranchese & Sugiura, 2021), hacker collectives (Décary-Hétu & Dupont, 2012) or other extremist online communities. Promising steps toward this work have already determined that fusion can be detected in potentially dangerous online communities, such as QAnon, via systematic linguistic analyses (Ebner et al., 2022a, b). Further, Rousis et al. (2023) confirmed that incels are more fused to the ingroup compared to men active in other male-dominated groups and that these highly fused men were more likely to engage in violence and harassment against women.

An as yet underexplored dimension in the study of identity fusion is the role of subcultural, contextual, and structural factors—particularly class—in shaping pathways toward fusion. In contrast criminological research has long recognised that individuals’ embeddedness within subcultures plays a significant role in shaping behavioural trajectories, including participation in deviant or criminal activities. Classic subcultural theories (Cohen, 1955; Cloward & Ohlin, 1960) argued that deviant subcultures emerge in response to blocked opportunities and structural disadvantages, providing alternative status systems and collective identities through which individuals seek recognition and belonging. More recent work in cultural criminology (Ferrell et al., 2015) further highlights the emotional and symbolic appeal of subcultures, framing them as spaces where individuals forge deep affective ties and shared moral frameworks that normalise risk-taking and transgression. Such findings are highly relevant for understanding identity fusion, as they suggest that pathways to fusion may also be deeply shaped by individuals’ socio-economic positions and cultural affiliations. How does fusion to social targets vary within class or cultural groups?

Research within crime science has also begun to recognise the importance of these contextual and structural factors in shaping offending patterns, particularly through the lens of situational action theory (Wikström, 2010), which emphasises the interaction between personal propensities and environmental settings. In this light, individuals from marginalised or deprived backgrounds may be especially susceptible to fusion with subcultures that valorise deviance, particularly where alternative sources of status, meaning, or social capital are lacking (Sandberg, 2008). Thus, the likelihood of fusion with criminogenic groups may vary systematically according to class, neighbourhood conditions, and social exclusion—factors often neglected in the more individualistic approaches to fusion theory rooted in social psychology. Many fusion studies employ social economic status as part of standard demographics, but to our knowledge a detailed study of structural factors’ relationship to the construction of fusion have not yet been published. Taken together, criminological understandings of subcultural and contextual factors suggests that efforts to prevent harmful forms of fusion must attend not only to group characteristics but also to the wider social and structural conditions that shape the availability and appeal of different fusion pathways.

### Fusion and reintegration

By understanding the role of fusion in criminal behaviour we may be better placed to devise strategies to support the reintegration of formerly incarcerated individuals post release and reduce rates of recidivism. On the one hand, fusion with law-abiding groups could help individuals to reduce their risks of reoffending and act in ways that benefit the community. On the other hand, fusion with formerly incarcerated individuals on the part of receiving communities could play a crucial role in successful reintegration.

Although fusion with a group is usually long-lasting, several psychological studies have pointed to instances where fusion to large-scale group identities has decreased (Vázquez et al., 2017b; Gómez et al., 2019). It is possible that a shift in fusion towards mainstream values and group identities could be achieved via key moments of re-evaluation. In Laub and Sampson’s (2003) research, they found that many former offenders who desisted from crime had experienced ‘turning points’ in their lives - periods of good employment, strong marriages or good relationships, periods in the army, or moving away from problematic neighbourhoods– and these turning points transform the sense of self. Put differently, (successful) turning points are moments of identity change, and fusion to conventional, pro-social roles (spouse, employer, military discipline, neighbourhood) can play a role in crystalising these changes. Fusion theory therefore not only complements but operationalises Sampson and Laub’s insight: desistance should be strongest when the emerging prosocial identity is fused, i.e., when personal and role identities become psychologically indistinguishable. Does fusion to alternative (more socially acceptable) groups, therefore, fully mediate the positive effect of marriage or employment (etc.)? Do interventions that aim to foster fusion to pro-social groups as part of these turning points lead to longer lasting and larger reductions in offending behaviour, and how can mainstream interventions provide access to meaningful alternative group identities?

Fostering more positive forms of fusion among formerly incarcerated individuals might realistically focus on activities that have street credibility and an inclusive and welcoming ethos. In some cases, activities might include minor forms of illegal behaviour or counter-cultural elements. For example, Newson et al. (2021) investigated how transformative experiences derived from taking psychedelic drugs in a ritualised environment (i.e., at an illegal rave) can lead to fusion. The research posited that such intense experiences can lead an individual to build meaningful and lasting social bonds with associated pro-group behavioural outcomes. The roles of intense, self-transformative experiences in facilitating identity fusion are well reported in various contexts, including Libyan insurgents, soccer fans, survivors of terrorist attacks, martial arts practitioners, and Northern Irish Republicans and Unionists (Whitehouse et al., 2014; Jong et al., 2015; Newson et al., 2016; Kavanagh et al., 2019).

More subtle processes where a (positive) group identity becomes internalised may play a role in desistance more generally, such as might be seen via restorative justice interventions. An example of this is the Twinning Project, a sports-based prison and probation intervention that pairs major soccer clubs in the UK with local prison services to deliver coaching, stewarding or refereeing programmes (Newson & Whitehouse, 2020). While these programmes offer accredited qualifications, they also provide meaningful and social identities, with associated behavioural improvements (Newson et al., 2024c). The premise is that the soccer ‘family’ is a powerful enough identity for formerly incarcerated individuals to modify their behaviours long term, if the group identity is internalised, i.e., when they become fused to the target.

Marshall (1999) argues that a fully restorative justice resolution involves the offender, victim and, crucially, the community - however difficult that can be to define. Researchers have long appreciated that the prospects of rehabilitation and reduced rates of recidivism are heavily influenced by the response of the receiving community, including the willingness of employers to offer ‘second chances’ to applicants with criminal records (Caspi & Moffitt, 1993; Laub & Sampson, 2003; Petersilia, 2003; Bersani et al., 2009; Craig & Foster, 2011; Kirk, 2012; Skardhamar & Savolainen, 2014). There is some evidence that when prospective employers reflect on the sufferings endured by formerly incarcerated individuals, it can activate the shared-experiences-pathway to fusion and increase willingness to offer employment opportunities (Peitz et al., 2025). Following the same logic, community organisations, religious congregations, clubs and societies, and other law-abiding groups could be encouraged to play a greater role helping formerly incarcerated individuals to become re-established in the community as a result of interventions aimed at fostering fusion in ways that motivate support for reintegration efforts. The Twinning Project mentioned above is adopting this approach through its work with soccer clubs and local prisons but similar approaches could be adapted to a much wider range of sports clubs and other comparable associations. The advantages of interventions targeting subject identity through the fusion lens poses exciting avenues for future research.

There is also a potentially important role for research into the role of the media in tackling crime and reducing rates of recidivism. Salacious media reporting of violent crimes can inflate perceptions of public risk and increase demand for punitive public policy and increased incarceration rates, even when the evidence suggests that this is not the most effective way to reduce crime (Enns, 2014; Matthews, 2005; Newburn, 2007). Lacking is research into the kinds of media reporting that could foster higher rates of fusion towards those currently ensnared in preventable cycles of criminal activity, in ways that motivate more effective and lasting reintegration programmes. Relatedly, victim support groups could do more to foster fusion among those who have been harmed by crime in ways that can support campaigns for restorative justice that assist rehabilitation and reduce crime rates.

## Future directions and conclusions

The fusion framework has the potential to be a powerful analytical tool, both in terms of understanding the role it plays in mediating offending behaviour, but also how it can be leveraged to contribute to the policy toolkit to counteract offending behaviours. Importantly, fused people should not be labelled as ‘criminal’, rather understanding how people become fused and why they behave in extremes could help inform best practice in designing interventions to decrease criminal behaviours. The knowledge and theorising that crime researchers can bring to understanding these, and other, behaviours will aid the study of fusion, as much as the conceptualisation of certain types of extreme bonding as represented by identity fusion will be able to aid aspects of crime science more broadly.

Importantly, the ‘pro-group’ behaviours commonly associated with identity fusion are not inherently ‘positive’ or ‘negative’, rather they are in the best interest of the group (Whitehouse, 2018), whether it is violence in the name of the group (Newson et al., 2018) or pro-sociality such as stopping to help someone out, buying them a drink, or giving them a hug (Newson et al., 2022). Similarly, the events that it can cause or protect against are not universal and need to be considered within the context of what identity fusion to any given group or value is likely to affect. Table 1 offers tentative areas that the partnership between fusion research and criminological inquiry could tackle, enriching both fields simultaneously. This is not a definitive list and merely what appears to us as the most fruitful avenues for immediate research and theorising. Conceptualising social harms (i.e., zemiology) is foundational to criminology; something that could help develop fusion theory to better label and classify fusion-associated behaviours.

To date, little research has utilised research on fusion from a policy perspective (the example of research on the Twinning Project mentioned above notwithstanding). There is also emerging evidence that fusion theory could contribute to the development of interventions that could reduce or ameliorate the effects of fusion to criminal groups - such as the work by Gómez et al. (2021) which identified admiration as a key determinant of the fusion process of Jihadist to radical Islamist groups. By identifying the processes that lead to fusion practitioners can build models that enhance the positive effects of fusion on positive forms of prosocial action and find ways to counteract the negative effects of fusion among antisocial and criminal groups. Going forward, criminological and sociological contributions are greatly needed to help establish who has the power to decide which forms of fusion and which behaviours are classed as ‘positive’, ‘negative’, or ‘antisocial’: fusion theory is rooted in anthropology and psychological positivist traditions that lack the philosophical resources to address issues of ‘right and wrong’ that are so central to constructivist traditions.

This article has sought to introduce the value of integrating identity fusion theory with crime science research and practice. By demonstrating the applicability of fusion theory in the dynamics of offending behaviour, we advance three key contributions. First, we contribute to one of criminology’s most enduring debates: the contested nature of social bonds and their relationship to antisocial behaviour. By bridging insights from control theory and social learning theory, we demonstrate how identity fusion complicates dichotomies regarding whether deviant behaviour is caused by the absence of social bonds or their presence within delinquent groups. Rather than viewing social ties as either protective or criminogenic, the fusion framework adds to a nuanced understanding of how strong relational ties can facilitate both prosocial and antisocial actions, depending on group norms and contextual factors.

Second, this article offers a different conceptual perspective on the role of identity processes in shaping criminal behaviour. While much research within crime science has focused on external social structures and peer dynamics, our approach highlights the importance of internalised social identities—particularly fused identities—in motivating extreme behaviours. Crucially, we challenge characterisations of ‘fused’ individuals as inherently criminal or dangerous. Instead, we argue that a deeper understanding of the processes through which people become fused, and the conditions under which fusion yields harmful or prosocial behaviours, has substantial implications for theory, intervention, and policy design. In doing so, we encourage criminologists to engage with our emerging research on identity processes, while urging fusion researchers to consider the complexities of social harms, moral frameworks, and structural inequalities that criminology routinely addresses.

Third, this article bridges academic silos by bringing together theoretical and empirical insights from criminology, anthropology, psychology, and sociology—fields that have too often developed in isolation. By synthesising these perspectives, we have identified blind spots that limit the explanatory power of existing models of extreme behaviour. In particular, we argue that integrating fusion theory with criminological debates surrounding social bonds, moral agency, and group dynamics not only enriches theoretical development, but also initiates a process of triangulating evidence across disciplines to address complex social problems more effectively. We hope this integrative approach will stimulate future research that crosses disciplinary boundaries to address difficult questions about violence, group loyalty, and prosociality, while also offering new tools for practitioners seeking to mitigate the harmful effects of fusion in criminogenic contexts.

Ultimately, we position this article as a call for greater dialogue between crime science and fusion research, advocating for more systematic collaboration in theorising, empirical testing, and policy engagement. By doing so, we aim to contribute to the development of more refined and context-sensitive approaches to understanding, preventing, and responding to both antisocial and prosocial forms of extreme group behaviour.

## Data Availability

Not applicable. There is no data pertaining to this article.

## References

[CR1] Agnew, R. (1992). Foundation for a general strain theory of crime and delinquency. *Criminology*, *30*(1), 47–88. 10.1111/j.1745-9125.1992.tb01093.x

[CR2] Agnew, R. (2006). *Pressured into crime: An overview of general strain theory*. Roxbury.

[CR3] Akers, R. L. (1998). *Social learning and social structure: A general theory of crime and deviance*. Northeastern University.

[CR4] Akers, R. (2017). *Social learning and social structure: A general theory of crime and deviance*. Routledge. 10.4324/9781315129587

[CR5] Apps, M. A., McKay, R., Azevedo, R. T., Whitehouse, H., & Tsakiris, M. (2018). Not on my team: Medial prefrontal cortex responses to ingroup fusion and unfair monetary divisions. *Brain and Behavior*, *8*(8), 1–13. 10.1002/brb3.1030 10.1002/brb3.1030 PMC6085923 29931824

[CR6] Aron, A., Aron, E. N., & Smollan, D. (1992). Inclusion of other in the self scale and the structure of interpersonal closeness. *Journal of Personality and Social Psychology*, *63*(4), 596–612. 10.1037/0022-3514.63.4.596

[CR7] Becker, H. S. (1963). *Outsiders: Studies in the sociology of deviance*. Free Press Glencoe.

[CR8] Bersani, B., Laub, J. H., & Nieuwbeerta, P. (2009). Marriage and desistance from crime in the netherlands: Do gender and socio-historical context matter? *Journal of Quantitative Criminology*, *25*(1), 3–24. 10.1007/s10940-008-9056-4

[CR9] Bortolini, T., Bado, P., Hoefle, S., Engel, A., Zahn, R., de Oliveira Souza, R., Dreher, J., & Moll, J. (2017). Neural bases of ingroup altruistic motivation in football fans. *Scientific Reports*, *7*(1), 1–12. 10.1038/s41598-017-15385-7 29170383 10.1038/s41598-017-15385-7 PMC5700961

[CR10] Bortolini, T., Newson, M., Natividade, J. C., Vázquez, A., & Gómez, Á. (2018). Identity fusion predicts endorsement of pro-group behaviours targeting nationality, religion, or rootball in Brazilian samples. *British Journal of Social Psychology*, *57*(2), 346–366. 10.1111/bjso.12235 29322509 10.1111/bjso.12235

[CR11] Braithwaite, J. (1989). *Crime, shame and reintegration*. Cambridge University Press.

[CR12] Buhrmester, M. D., Fraser, W. T., Lanman, J. A., Whitehouse, H., & SwannJr., W. B. (2015). When terror hits home: Identity fused Americans who saw Boston bombing victims as family provided aid. *Self and Identity*, *14*(3), 253–270. 10.1080/15298868.2014.992465

[CR13] Buhrmester, M. D., Zeitlyn, D., & Whitehouse, H. (2022). Ritual, fusion, and conflict: The roots of agro-pastoral violence in rural Cameroon. *Group Processes & Intergroup Relations*, *25*(1), 298–311. 10.1177/1368430220959705

[CR14] Burgess, R. L., & Akers, R. L. (1966). A differential association-reinforcement theory of criminal behavior. *Social Problems*, *14*(2), 128–147. 10.1525/sp.1966.14.2.03a00020

[CR15] Caspi, A., & Moffitt, T. E. (1993). When do individual differences matter? A Paradoxical theory of personality coherence. *Psychological Inquiry*, *4*(4), 247–271. 10.1207/s15327965pli0404_1

[CR16] Chinchilla, J., Vazquez, A., & Gómez, Á. (2022). Identity fusion predicts violent pro-groupbehavior when it is morally justifiable. *The Journal of Social Psychology*, *162*(6), 701–715. 10.1080/00224545.2021.1948813 34353239 10.1080/00224545.2021.1948813

[CR17] Cloward, R. A., & Ohlin, L. E. (1960). *Delinquency and opportunity: A theory of delinquent gangs*. Free.

[CR18] Cohen, A. K. (1955). *Delinquent boys: The culture of the gang*. Free.

[CR19] Craig, J., & Foster, H. (2011). Desistance in the transition to adulthood: The roles of marriage, military and gender. *Deviant Behavior*, *34*(3), 208–223. 10.1080/01639625.2012.726173

[CR20] Décary-Hétu, D., & Dupont, B. (2012). The social network of hackers. *Global Crime Volume*, *13*(3), 160–175. 10.1080/17440572.2012.702523

[CR21] Ebner, J., Kavanagh, C., & Whitehouse, H. (2022a). Is there a Language of terrorists? A comparative manifesto analysis. *Studies in Conflict & Terrorism*, 1–27. 10.1080/1057610X.2022.2109244

[CR22] Ebner, J., Kavanagh, C., & Whitehouse, H. (2022b). The QAnon security threat: A linguistic fusion-based violence risk assessment. *Perspectives on Terrorism*, *16*(6), 62–86. https://www.jstor.org/stable/27185092

[CR23] Ebner, J., Kavanagh, C., & Whitehouse, H. (2024). Assessing violence risk among far-right extremists: A new role for natural Language processing. *Terrorism and Political Violence*, *36*(7), 944–961. 10.1080/09546553.2023.2236222 39257630 10.1080/09546553.2023.2236222 PMC11382783

[CR24] Enns, P. K. (2014). The public’s increasing punitiveness and its influence on mass incarceration in the united States. *American Journal of Political Science*, *58*(4), 857–872. 10.1111/ajps.12098

[CR26] Farrington, D. P. (2007). Advancing knowledge about desistance. *Journal of Contemporary Criminal Justice*, *23*(1), 125–134. 10.1177/1043986206298954

[CR25] Farrington, D. P., & Welsh, B. C. (2006). *Saving children from a life of crime: Early risk factors and effective interventions*. Oxford University Press. 10.1093/acprof:oso/9780195304091.001.0001

[CR27] Federal Bureau of Investigation (2011). *2011 National Gang Threat Assessment*. Federal Bureau of Investigation. https://www.fbi.gov/stats-services/publications/2011-national-gang-threat-assessment. Accessed 15 January 2025.

[CR28] Ferrell, J., Hayward, K., & Young, J. (2015). *Cultural criminology: An invitation* (2nd ed.). Sage.

[CR29] Fredman, L. A., Bastian, B., & SwannJr., W. B. (2017). God or country? Fusion with Judaism predicts desire for retaliation following Palestinian stabbing intifada. *Social Psychological and Personality Science*, *8*(8), 882–887. 10.1177/1948550617693059

[CR30] Gómez, Á., Brooks, M. L., Buhrmester, M. D., Vázquez, A., Jetten, J., & Swann, W. B. Jr. (2011). On the nature of identity fusion: Insights into the construct and a new measure. *Journal of Personality and Social Psychology*, *100*(5), 918–933. 10.1037/a0022642 21355659 10.1037/a0022642

[CR31] Gómez, A., Vázquez, A., López-Rodríguez, L., Talaifar, S., Martinez, M., Buhrmester, M. D., & Swann, W. B. Jr. (2019). Why people abandon groups: Degrading relational vs. collective ties uniquely impacts identity fusion and identification. *Journal of Experimental Social Psychology*, *85*, 103853. 10.1016/j.jesp.2019.103853

[CR32] Gómez, A., Chinchilla, J., Vázquez, A., López-Rodríguez, L., Paredes, B., & Martínez, M. (2020). Recent advances, misconceptions, untested assumptions, and future research agenda for identity fusion theory. *Social and Personality Psychology Compass*, *14*(6), 1–15. 10.1111/spc3.12531

[CR33] Gómez, Á., Bélanger, J. J., Chinchilla, J., Vázquez, A., Schumpe, B. M., Nisa, C. F., & Chiclana, S. (2021). Admiration for islamist groups encourages self-sacrifice through identity fusion. *Humanities and Social Sciences Communications*, *8*(1), 1–12. 10.1057/s41599-021-00734-938617731

[CR34] Gottfredson, M. R., & Hirschi, T. (1990). *A general theory of crime*. Stanford University Press.

[CR35] Haslam, S. A., Jetten, J., Postmes, T., & Haslam, C. (2009). Social identity, health and well-being: An emerging agenda for applied psychology. *Applied Psychology*, *58*(1), 1–23. 10.1111/j.1464-0597.2008.00379.x

[CR36] Hirschi, T. (2002). *Causes of delinquency*. University of California Press.

[CR37] Jiménez, J., Gómez, Á., Buhrmester, M. D., Vázquez, A., Whitehouse, H., & Swann, W. B. (2016). The dynamic identity fusion index: A new continuous measure of identity fusion for web-based questionnaires. *Social Science Computer Review*, *34*(2), 215–228. 10.1177/0894439314566178

[CR38] Jolliffe, D., & Farrington, D. P. (2004). Empathy and offending: A systematic review and meta-analysis. *Aggression and Violent Behavior*, *9*(5), 441–476. 10.1016/j.avb.2003.03.001

[CR39] Jolliffe, D., & Farrington, D. P. (2006). Examining the relationship between low empathy and bullying. *Aggressive Behavior*, *32*(6), 540–550. 10.1002/ab.20154

[CR40] Jolliffe, D., & Farrington, D. P. (2010). Individual differences and offending. In R. Newburn, & E. McLaughlin (Eds.), *The SAGE handbook of criminological theory*. Sage.

[CR41] Jong, J., Whitehouse, H., Kavanagh, C., & Lane, J. (2015). Shared negative experiences lead to identity fusion via personal reflection. *PloS One*, *10*(12), 1–12. 10.1371/journal.pone.0145611 10.1371/journal.pone.0145611 PMC4689389 26699364

[CR42] Joo, M., & Park, S. W. (2017). Effect of identity fusion on decision to make extreme sacrifices in romantic relationships: The moderating role of impulsiveness. *British Journal of Social Psychology*, *56*(4), 819–827. 10.1111/bjso.12218 28895158 10.1111/bjso.12218

[CR43] Kavanagh, C. M., Jong, J., McKay, R., & Whitehouse, H. (2019). Positive experiences of high arousal martial arts rituals are linked to identity fusion and costly pro-group actions. *European Journal of Social Psychology*, *49*(3), 461–481. 10.1002/ejsp.2514 31598015 10.1002/ejsp.2514 PMC6774318

[CR44] Kirk, D. S. (2012). Residential change as a turning point in the life course of crime: Desistance or temporary cessation? *Criminology*, *50*(2), 329–358. 10.1111/j.1745-9125.2011.00262.x

[CR45] Klein, J. W., & Bastian, B. (2022). The fusion-secure base hypothesis. *Personality and Social Psychology Review*, *27*(2), 107–127. 10.1177/1088868322110088 35708063 10.1177/10888683221100883

[CR46] Kunst, J. R., Boos, B., Kimel, S. Y., Obaidi, M., Shani, M., & Thomsen, L. (2018). Engaging in extreme activism in support of others’ political struggles: The role of politically motivated fusion with out-groups. *PloS One*, *13*(1), 1–30. 10.1371/journal.pone.0190639 10.1371/journal.pone.0190639 PMC5755793 29304156

[CR47] Kunst, J. R., Dovidio, J. F., & Thomsen, L. (2019). Fusion with political leaders predicts willingness to persecute immigrants and political opponents. *Human Behaviour*, *3*(11), 1180–1189. 10.1038/s41562-019-0708-1 10.1038/s41562-019-0708-1 31477913

[CR48] Landabur, R., Miguez, G., Laborda, M. A., & Salinas, M. I. (2022). Why do people self-sacrifice for their country? The roles of identity fusion and empathic concern. *PsyCh Journal*, *11*(1), 55–64. 10.1002/pchj.495 34749442 10.1002/pchj.495

[CR49] Laub, J. H., & Sampson, R. J. (2003). *Shared beginnings, divergent lives: Delinquent boys to age 70*. Harvard University Press.

[CR50] Lemert, E. M. (1951). *Social pathology: A systematic approach to the theory of sociopathic behavior*. McGraw-Hill.

[CR51] Lin, J. S., & Sung, Y. (2014). Nothing can tear Us apart: The effect of brand identity fusion in consumer–vrand relationships. *Psychology & Marketing*, *31*(1), 54–69. 10.1002/mar.20675

[CR52] Marshall, T. F. (1999). *Restorative justice: An overview. A report by the home office research, development and statistics directorate*. Home Office.

[CR53] Mason, C. B., Winter, D. A., Schmeer, S., & Bell, R. C. (2025). Radicalized Trump supporters: Construing, identity fusion, and hypothetical and actual extremism. *Journal of Constructivist Psychology*, *38*(1), 89–118. 10.1080/10720537.2024.2394075

[CR54] Matthews, R. (2005). The myth of punitiveness. *Theoretical Criminology*, *9*(2), 175–201. 10.1177/1362480605051639

[CR55] McAra, L., & McVie, S. (2012). Negotiated order: The groundwork for a theory of offending pathways. *Criminology & Criminal Justice*, *12*(4), 347–375. 10.1177/1748895812455810

[CR56] Misch, A., Fergusson, G., & Dunham, Y. (2018). Temporal dynamics of partisan identity fusion and prosociality during the 2016 US presidential election. *Self and Identity*, *17*(5), 531–548. 10.1080/15298868.2018.1430063

[CR57] Newburn, T. (2007). Tackling youth grime and reforming youth justice: The origins and nature of ‘new labour’ policy. *Policy Studies*, *19*(3–4), 199–212. 10.1080/01442879808423755

[CR60] Newson, M. (2019). Football, fan violence, and identity fusion. *International Review for the Sociology of Sport*, *54*(4), 431–444. 10.1177/1012690217731293

[CR61] Newson, M., & Whitehouse, H. (2020). The twinning project: How football, the beautiful game, can be used to reduce reoffending. *Prison Service Journal*, *248*, 28–31.

[CR58] Newson, M., Buhrmester, M., & Whitehouse, H. (2016). Explaining lifelong loyalty: The role of identity fusion and self-shaping group events. *PloS One*, *11*(8). 10.1371/journal.pone.0160427 10.1371/journal.pone.0160427 PMC4980014 27508386

[CR59] Newson, M., Bortolini, T., Buhrmester, M., da Silva, S., Acquino, J., & Whitehouse, H. (2018). Brazil’s football warriors: Social bonding and inter-group violence. *Evolution & Human Behavior*, *39*(6). 10.1016/j.evolhumbehav.2018.06.010

[CR62] Newson, M., Khurana, R., Cazorla, F., & van Mulukom, V. (2021). I get high with a little help from my friends’—how raves can invoke identity fusion and lasting co-operation via transformative experiences. *Frontiers in Psychology*, *12*. 10.3389/FPSYG.2021.719596/BIBTEX 10.3389/fpsyg.2021.719596 PMC8504457 34646208

[CR63] Newson, M., White, F., & Whitehouse, H. (2022). Does loving a group mean hating its rivals? Exploring the relationship between ingroup cohesion and outgroup hostility among football fans. *International Journal of Sport and Exercise Psychology*, *21*(4), 706–724. https://doi.org/0.1080/1612197X.2022.2084140

[CR64] Newson, M., Peitz, L., Wibisono, S., Knijnik, J., White, F., & Whitehouse, H. (2024a). Anti-social behavior and soccer identities: Different continents, same mindset? *Self and Identity*, *23*(7–8), 616–633. 10.1080/15298868.2024.2423829 39659800 10.1080/15298868.2024.2423829 PMC11627210

[CR65] Newson, M., Peitz, L., Gitsham, H., Imada, H., & Abrams, D. (2024b). We need community’: Bridging the path to desistance from crime with community football. *Journal of Community and Applied Social Psychology*, 34. 10.1002/casp.2757

[CR66] Newson, M., Peitz, L., Cunliffe, J., & Whitehouse, H. (2024c). A soccer-based intervention improves incarcerated individuals’ behaviour and public acceptance through group bonding. *Nature Human Behaviour*, *8*(12), 2304–2313. 10.1038/s41562-024-02006-3 10.1038/s41562-024-02006-3 PMC11659165 39402257

[CR67] Ozkan, Z., El-Astal, S., & Cakal, H. (2025). Paths to peaceful and violent action: Identity fusion and group identification. *British Journal of Social Psychology*, *64*(3), e70002. 10.1111/bjso.70002 40542523 10.1111/bjso.70002 PMC12181741

[CR68] Paredes, B., Briñol, P., & Gómez, Á. (2018). Identity fusion leads to willingness to fight and die for the group: The moderating impact of being informed of the reasons behind other members’ sacrifice. *Self and Identity*, *17*(5), 517–530. 10.1080/15298868.2017.1419503

[CR69] Paternoster, R. (2017). Happenings, acts, and actions: Articulating the meaning and implications of human agency for criminology. *Journal of Developmental and Life-Course Criminology*, *3*(4), 350–372. 10.1007/s40865-017-0069-2

[CR70] Peitz, L., Whitehouse, H., & Newson, M. (2025). Can transformative experiences Bridge the gap between receiving communities and formerly incarcerated persons? *British Journal of Social Psychology*, *64*(3), e12886. 10.1111/bjso.12886 40326550 10.1111/bjso.12886 PMC12053958

[CR71] Petersilia, J. (2003). *When prisoners come home: Parole and prisoner reentry*. Oxford University Press.

[CR72] Piquero, A. R., Jennings, W. G., & Farrington, D. P. (2010). On the malleability of Self-Control: Theoretical and policy implications regarding a general theory of crime. *Justice Quarterly*, *27*(6), 803–834. 10.1080/07418820903379628

[CR73] Pyrooz, D. C., & Decker, S. H. (2019). *Competing for control: Gangs and the social order of prisons*. Cambridge University Press. 10.1017/9781108653473

[CR74] Reese, E., & Whitehouse, H. (2021). The development of identity fusion. *Perspectives on Psychological Science*, *16*(6), 1398–1411. 10.1177/1745691620968761 33684334 10.1177/1745691620968761

[CR75] Reicher, S. (2001). The psychology of crowd dynamics. In M. A. Hogg, & R. S. Tindale (Eds.), *Blackwell handbook of social psychology: Group processes* (pp. 182–208). Blackwell Publishers Ltd.

[CR76] Rosenfeld, R., Jacobs, B. A., & Wright, R. (2003). Snitching and the code of the street. *The British Journal of Criminology*, *43*(2), 291–309. 10.1093/bjc/43.2.291

[CR77] Rousis, G. J., Martel, F. A., Bosson, J. K., & SwannJr., W. B. (2023). Behind the blackpill: Self-verification and identity fusion predict endorsement of violence against women among self-identified incels. *Personality and Social Psychology Bulletin*, *50*(11), 1531–1545. 10.1177/01461672231166481 37070745 10.1177/01461672231166481

[CR78] Sandberg, S. (2008). Street capital: Ethnicity and violence on the streets of Oslo. *Theoretical Criminology*, *12*(2), 153–171. 10.1177/1362480608089238

[CR79] Sheikh, H., Gómez, Á., & Atran, S. (2016). Empirical evidence for the devoted actor model. *Current Anthropology*, *57*(S13), S204–S209. 10.1086/686221

[CR80] Skardhamar, T., & Savolainen, J. (2014). Changes in criminal offending around the time of job entry: A study of employment and desistance. *Criminology*, *52*(2), 263–291. 10.1111/1745-9125.12037

[CR81] Smith, W., & Lewis, M. (2022). *Both/And thinking: Embracing creative tensions to solve your toughest problems*. Harvard Business Review.

[CR83] Stott, C., & Pearson, G. (2007). *Soccer ‘hooliganism’: Policing and the war on the ‘English disease’*. Pennant Books.

[CR82] Stott, C., Hutchison, P., & Drury, J. (2001). Hooligans’ abroad? Inter-group dynamics, social identity and participation in collective ‘disorder’ at the 1998 world cup finals. *British Journal of Social Psychology*, *40*(3), 359–384. 10.1348/014466601164876 11593939 10.1348/014466601164876

[CR84] Stott, C., Adang, O., Livingstone, A., & Schreiber, M. (2008). Tackling soccer hooliganism: A quantitative study of public order, policing and crowd psychology. *Psychology Public Policy and Law*, *14*(2), 115. 10.1037/a0013419

[CR85] Stott, C., Havelund, J., Lundberg, F., Khan, S., Joern, L., Hogget, J., Rasmussen, K., & Vestergren, S. (2016). Policing soccer in Sweden: enabling an evidence based approach. https://www.ucviden.dk/en/publications/politiets-h%C3%A5ndtering-af-fodboldsupportere-i-sverige-en-evidensbas. Accessed 15 January 2025.

[CR86] Stott, C., Havelund, J., & Williams, N. (2019). Policing soccer crowds in Sweden. *Nordic Journal of Criminology*, *20*(1), 35–53. 10.1080/14043858.2018.1513679

[CR87] Stott, C., Khan, S., Madsen, E., & Havelund, J. (2020). The value of supporter liaison officers (SLOs) in fan dialogue, conflict, governance and soccer crowd management in Sweden. *Soccer & Society*, *21*(2), 196–208. 10.1080/14660970.2018.1553777

[CR88] Sutherland, E. H. (1992 [1947]). *Principles of criminology* (4th ed.). J.B. Lippincott Company.

[CR93] SwannJr., W. B., & Buhrmester, M. D. (2015). Identity fusion. *Current Directions in Psychological Science*, *24*(1), 52–57. 10.1177/0963721414551363

[CR94] SwannJr., W. B., & Talaifar, S. (2018). Introduction to special issue of self and identity on identity fusion. *Self and Identity*, *17*(5), 483–486. 10.1080/15298868.2018.1458646

[CR89] SwannJr., W. B., Gómez, Á., Conor, S. D., Francisco, M. J., & Carmen, H. (2009). Identity fusion: The interplay of personal and social identities in extreme group behavior. *Journal of Personality and Social Psychology*, *96*(5), 995–1011. 10.1037/a0013668 19379032 10.1037/a0013668

[CR90] SwannJr., W. B., Jetten, J., Gómez, Á., Whitehouse, H., & Bastian, B. (2012). When group membership gets personal: A theory of identity fusion. *Psychological Review*, *119*(3), 441–456. 10.1037/a0028589 22642548 10.1037/a0028589

[CR91] SwannJr., W. B., Buhrmester, M. D., Gómez, A., Jetten, J., Bastian, B., Vázquez, A., Ariyanto, A., Besta, T., Christ, O., Cui, L., Finchilescu, G., González, R., Goto, N., Hornsey, M., Sharma, S., Susianto, H., & Zhang, A. (2014). What makes a group worth dying for? Identity fusion fosters perception of Familial ties, promoting self-sacrifice. *Journal of Personality and Social Psychology*, *106*, 912–926. 10.1037/a0036089 24841096 10.1037/a0036089

[CR92] SwannJr., W. B., Gómez, Á., Vázquez, A., Guillamón, A., Segovia, S., & Carillo, B. (2015). Fusion with the cross-gender group predicts genital sex reassignment surgery. *Archives of Sexual Behavior*, *44*(5), 1313–1318. 10.1007/s10508-014-0470-4 25666854 10.1007/s10508-014-0470-4

[CR96] Tajfel, H., & Turner, J. C. (1979). An integrative theory of intergroup conflict. In W. G. Austin, & S. Worchel (Eds.), *The social psychology of intergroup relations* (pp. 33–47). Brooks/Cole.

[CR97] Tasuji, T., Reese, E., van Mulukom, V., & Whitehouse, H. (2020). Band of mothers: Childbirth as a female bonding experience. *PloS One*, *15*(10). 10.1371/journal.pone.0240175 10.1371/journal.pone.0240175 PMC7577500 33085666

[CR98] Tittle, C. R. (1995). *Control balance: Toward a general theory of deviance*. Routledge.

[CR99] Tornero, E., Sánchez-Romera, J. F., Morosoli, J. J., Vázquez, A., Gómez, Á., & Ordoñana, J. R. (2017). Altruistic behavior among twins. *Human Nature*, *29*(1), 1–12. 10.1007/s12110-017-9304-0 10.1007/s12110-017-9304-0 29080969

[CR100] Tranchese, A., & Sugiura, L. (2021). I don’t hate all women, just those stuck-up bitches’: Howincels and mainstream pornography speak the same extreme Language of misogyny. *Violence against Women*, *27*(14), 2709–2734. 10.1177/1077801221996453 33750244 10.1177/1077801221996453 PMC8474329

[CR101] Tunçgenç, B., van Mulukom, V., & Newson, M. (2023). Social bonds are related to health behaviors and positive well-being globally. *Science Advances*, *9*(2). 10.1126/sciadv.add3715 10.1126/sciadv.add3715 PMC10957100 36638167

[CR102] Varmann, A. H., Kruse, L., Bierwiaczonek, K., Gómez, Á., Vázquez, A., & Kunst, J. R. (2023). How identity fusion predicts extreme pro-group orientations: A meta-analysis. *European Review of Social Psychology*, *35*(1), 162–197. 10.1080/10463283.2023.2190267

[CR103] Vázquez, A., Gómez, Á., Ordoñana, J., Swann, W., & Whitehouse, H. (2017a). Sharing genes fosters identity fusion and altruism. *Self and Identity*, *16*(6), 684–702. 10.1080/15298868.2017.1296887

[CR104] Vázquez, A., Gómez, Á., & SwannJr., W. B. (2017b). Do historic threats to the group diminish identity fusion and its correlates? *Self and Identity*, *16*(4), 480–503. 10.1080/15298868.2016.1272485

[CR105] Vázquez, A., López-Rodríguez, L., Martínez, M., Atran, S., & Gómez, Á. (2020). Threat enhances aggressive inclinations among devoted actors via increase in their relative physical formidability. *Personality and Social Psychology Bulletin*, *46*(10), 1461–1475. 10.1177/0146167220907466 32163015 10.1177/0146167220907466

[CR106] Walsh, C. M., & Neff, L. A. (2018). We’re better when we blend: The benefits of couple identity fusion. *Self and Identity*, *17*(5), 587–603. 10.1080/15298868.2018.1430062

[CR107] White, J. L., Moffitt, T. E., Caspi, A., Bartusch, D. J., Needles, D. J., & Stouthamer-Loeber, M. (1994). Measuring impulsivity and examining its relationship to delinquency. *Journal of Abnormal Psychology*, *103*(2), 192–205. 8040489 10.1037//0021-843x.103.2.192

[CR108] White, F. A., Newson, M., Verrelli, S., & Whitehouse, H. (2021). Pathways to prejudice and outgroup hostility: Group alignment and intergroup conflict among football fans. *Journal of Applied Social Psychology*, *51*(7), 660–666. 10.1111/jasp.12773

[CR112] Whitehouse, H. (2018). Dying for the group: Towards a general theory of extreme self-sacrifice. *Behavioral and Brain Sci Ences*, *41*(e192), 1–62. 10.1017/S0140525X18000249 10.1017/S0140525X18000249 29409552

[CR113] Whitehouse, H., & Fitzgerald, R. (2020). Fusion and reform: The potential for identity fusion to reduce recidivism and improve reintegration. *Anthropology in Action*, *27*(1), 1–13. 10.3167/aia.2020.270101

[CR109] Whitehouse, H., & Lanman, J. A. (2014). The ties that bind us: Ritual, fusion, and identification. *Current Anthropology*, *55*(6), 674–695. 10.1086/678698

[CR110] Whitehouse, H., McQuinn, B., Buhrmester, M., & Swann, W. B. Jr. (2014). Brothers in arms: Libyan revolutionaries bond like family. *Proceedings of the National Academy of Sciences*, *111*(50), 17783–17785. 10.1073/pnas.1416284111 10.1073/pnas.1416284111 PMC4273349 25385591

[CR111] Whitehouse, H., Jong, J., Buhrmester, M. D., Gómez, Á., Bastian, B., Kavanagh, C. M., Newson, M., Matthews, M., Lanman, J. A., McKay, R., & Gavrilets, S. (2017). The evolution of extreme Cooperation via shared dysphoric experiences. *Scientific Reports*, *7*(1), 1–10. 10.1038/srep44292 28290499 10.1038/srep44292 PMC5349572

[CR114] Wikström, P. O. H. (2010). Explaining crime as moral actions. In S. Hitlin & S. Vaisey (Eds.), *Handbook of the sociology of morality* (pp. 211–239). Springer. 10.1007/978-1-4419-6896-8_12

[CR115] Wolfowicz, M., Litmanovitz, Y., Weisburd, D., & Hasisi, B. (2021). Cognitive and behavioral radicalization: A systematic review of the putative risk and protective factors. *Campbell Systematic Reviews*, *17*(3), 1–90. 10.1002/cl2.1174 10.1002/cl2.1174 PMC10121227 37133261

